# Basic clinical examination of a patient with neuro-ophthalmology symptoms

**Published:** 2017-03-03

**Authors:** Anand Moodley

**Affiliations:** 1 Consultant neurologist Greys Hospital and University of KwaZulu-Natal Pietermaritzburg, South Africa.

**This article discusses how to clinically assess the visual pathway, examine the optic disc, check the pupil light reflexes and assess the extraocular movements in patients presenting with visual loss and/or diplopia.**

**Figure:**
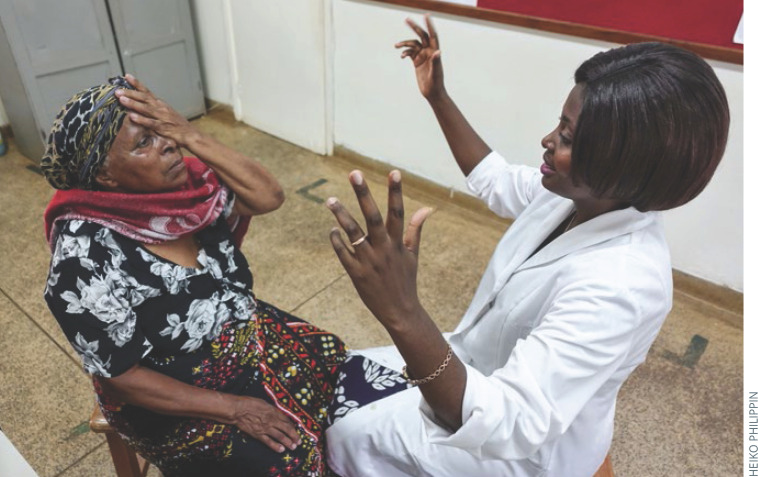
Testing a patient's visual fields by confrontation. TANZANIA

If a patient presents with potential neuro-ophthalmology signs and symptoms (see article on page 64), a basic neuro-ophthalmology examination should be undertaken. If done systematically, as described in this article, it can be informative in making a differential diagnosis and deciding on management.

A basic neuro-ophthalmological examination can be done with a minimum of equipment (see box below). Subsequent examination depends on what has been found and may involve full ocular and neurological examinations as well as investigations.

**Equipment required for a neuro-ophthalmological assessment**

- Snellen chart for far vision
- Pen torch for pupil reaction
- Occluder for cover testing
- Red probe or red mydriatic bottle top for colour desaturation testing
- Direct ophthalmoscope for fundoscopy

Also useful:

- Ruler for lid function and pupil diameter
- Cotton wool and office pin for sensation testing
- Pseudo-isochromatic plates for colour vision

**Figure 1.**
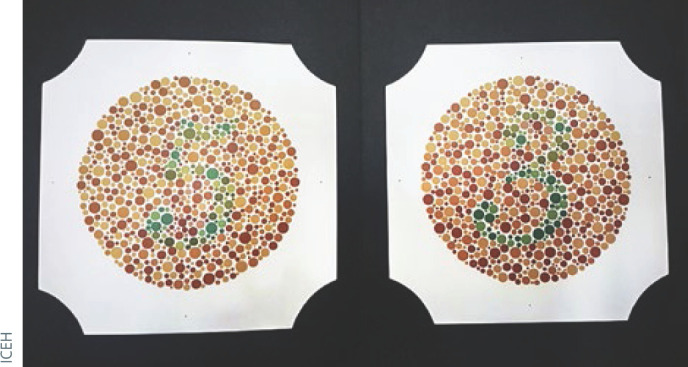
Ishihara pseudo-isochromatic plates for colour vision testing

Both the **visual pathway** and **oculomotor functioning** should be assessed.

## Visual pathway

### Visual acuity

The presenting and best corrected (with pinhole) visual acuity is obtained using a Snellen or LogMar chart for distance vision.

### Colour vision

Colour vision is tested using the Ishihara pseudo-isochromatic plates can be quantified by scoring the number of correct responses as an index of the total number of plates used (**Figure 1**). Such quantification allows for comparison during follow-up evaluations.

### Fundoscopy

Ophthalmoscopy is performed with particular attention to the optic disc, looking at possible swelling and the colour of the disc.

### Visual fields

**Figure 2.**
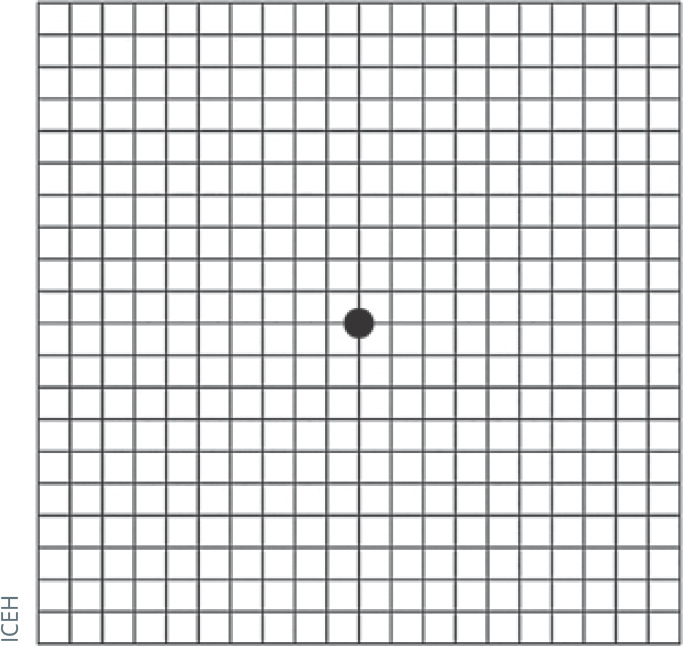
Amsler grid used for checking distortion of central vision

Visual field testing using a perimeter or, if not available, by confrontation, is done for each eye separately. Check the central vision with an Amsler grid (**Figure 2**) and then test the peripheral vision in each of the four quadrants.

***Read more:* www.cehjournal.org/article/visual-field-testing-for-glaucoma-a-practical-guide**

### Pupils

Examination of the pupil size, symmetry and reaction to a light stimulus provides information about both the afferent and efferent pathways.

Testing the pupil reflex in the dark is preferred as this makes it easier to appreciate constriction than in ambient room light.

A brisk, direct light reflex implies that the optic and 3rd nerve on that side are working well.

The swinging torch test is done by swinging the light source from one eye to the next while pausing for 3–5 seconds at each eye. It is used to detect a relative afferent pupillary defect (RAPD). This indicates that one optic nerve is not functioning as well as the other (the one that dilates as the torch light shines in it).

A dilated pupil that is non-reactive to a light source, together with ptosis, suggests a 3rd cranial nerve palsy; whereas a constricted, reactive pupil with partial ptosis suggests Homer syndrome from sympathetic impairment to the eye.

## Oculomotor functioning

### Ocular alignment

Check the alignment of the eyes. This is performed by comparing the light reflex from the cornea of both eyes. Hold a torch 1 metre in front of the eyes and look for the light reflex on the cornea (Hirschberg test). In the primary gaze (looking straight ahead at the torch light), the light reflexes should be in a symmetrical position on each cornea. If one eye is turning in, this is called esotropia, whereas if the eye is turning out it is called exotropia.

If you find that an eye is misaligned, use the cover test to confirm this. For example, say that you have observed the left eye turning in when both eyes look straight ahead. If you then cover the right eye (the normal eye), you should see the left eye (the deviated eye) turn out to take up fixation (i.e. look straight ahead).

If the left eye (in this example) does not realign when the other (normal) eye is covered, then the patient is either not cooperating or the eye is blind.

### Extraocular movements

**Figure 3.**
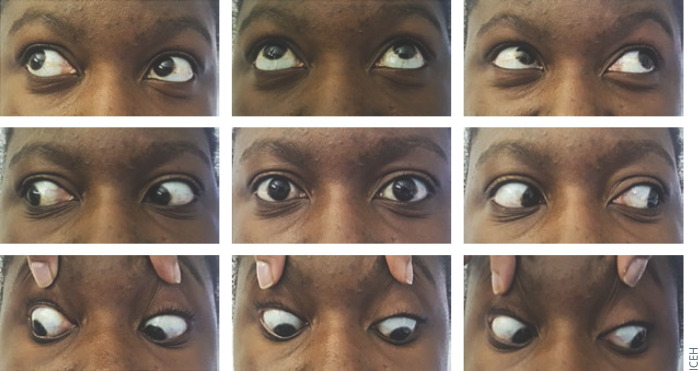
Testing of the 9 cardinal positions of the eyes

Test the ocular movements of one eye at a time in the 9 positions of gaze. Check for any limitation of movement (**Figure 3**).

Test the ocular movements of both eyes together to see if double vision is elicited in any position of gaze.

### Examination of eyelid movement

**Figure 4.**
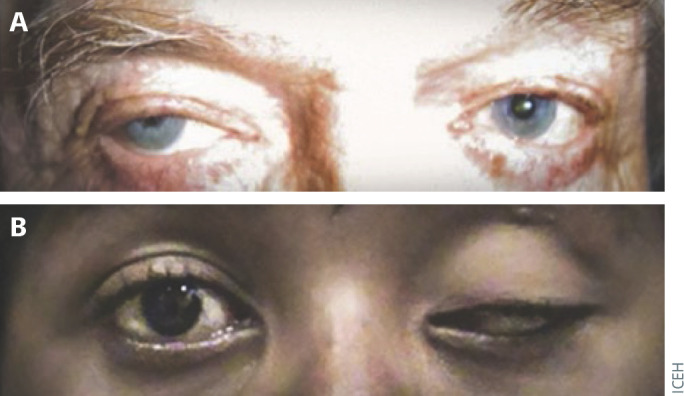
Examination of the eyelids: image A shows right-sided ptosis from Horner syndrome and image B shows left-sided ptosis from 3rd nerve palsy

Two muscles are responsible for opening the upper eyelid: the levator palpebrae superioris (supplied by the 3rd cranial nerve) and Muller's muscle (supplied by the sympathetic pathway). Hence, in 3rd cranial nerve palsies and Homer syndrome, ptosis may result (**Figure 4**).

The eyelids are closed by the orbicularis oculi muscle, which is supplied by the facial (7th) cranial nerve. In 7th nerve palsy, there is lagophthalmos (an inability to close the eye).

### Nystagmus

Nystagmus is rhythmical oscillations of the eyes. It may occur in the primary gaze or in horizontal or vertical gaze. Nystagmus has many causes, including ocular, vestibular and cerebellar conditions.

Jerk nystagmus is characterised by a slow ‘drift’ in one direction which is repeatedly corrected by a fast ‘recovery’ movement in the opposite direction.

Pendular nystagmus has equal speed in both directions and is commonly seen in congenital nystagmus.

**Examination of the eyes in an unconscious patient**

Examination of the eyes is important in the evaluation of the unconscious patient; it includes examination of the pupils and fundoscopy.

**Pupils**

Pupillary testing involves assessment of the pupil size and reaction to light.

- In an unconscious patient, normal size and normally reactive pupils may suggest a metabolic encephalopathy from kidney failure, liver failure or electrolyte abnormalities.
- A unilateral or bilateral dilated pupil can suggest 3rd nerve palsy due to herniation of the brain through the tentorium cerebelli. This is a surgical emergency.
- Bilateral pinpoint pupils that react to light may indicate pathology in the pons of the brain such as haemorrhage; or opioid or organophosphate poisoning.

**Fundoscopy**

- The presence of swollen optic discs suggest raised intracranial pressure.
- Preretinal haemorrhage may suggest ruptured intracranial blood vessels.
